# Silencing of Mcl-1 overcomes resistance of melanoma cells against TRAIL-armed oncolytic adenovirus by enhancement of apoptosis

**DOI:** 10.1007/s00109-021-02081-3

**Published:** 2021-05-24

**Authors:** Beatrice Tolksdorf, Sina Zarif, Jürgen Eberle, Ahmet Hazini, Babette Dieringer, Franziska Jönsson, Florian Kreppel, Jens Kurreck, Henry Fechner

**Affiliations:** 1 Department of Applied Biochemistry, Institute of Biotechnology, Technische Universität Berlin, Gustav-Meyer-Allee 25, 15533 Berlin, Germany; 2 Department of Dermatology, Venerology and Allergology, Skin Cancer Center Charité, Charité-Universitätsmedizin Berlin (University Medical Center Charité), 10117 Berlin, Germany; 3 Faculty of Health, School of Medicine, Center for Biomedical Research and Education ZBAF, Witten/Herdecke University, Stockumer Straße 10, 58453 Witten, Germany

**Keywords:** Apoptosis, Mcl-1, Melanoma, Oncolytic adenovirus, siRNA, TRAIL

## Abstract

**Abstract:**

Arming of oncolytic viruses with tumor necrosis factor-related apoptosis-inducing ligand (TRAIL) has been shown as a viable approach to increase the antitumor efficacy in melanoma. However, melanoma cells may be partially or completely resistant to TRAIL or develop TRAIL resistance, thus counteracting the antitumor efficiency of TRAIL-armed oncolytic viruses. Recently, we found that TRAIL resistance in melanoma cells can be overcome by inhibition of antiapoptotic Bcl-2 protein myeloid cell leukemia 1 (Mcl-1). Here, we investigated whether the cytotoxicity of AdV-TRAIL, an oncolytic adenovirus, which expresses TRAIL after induction by doxycycline (Dox), can be improved in melanoma cells by silencing of Mcl-1. Two melanoma cell lines, the TRAIL-resistant MeWo and the TRAIL-sensitive Mel-HO were investigated. Treatment of both cell lines with AdV-TRAIL resulted in a decrease of cell viability, which was caused by an increase of apoptosis and necrosis. The proapoptotic effects were dependent on induction of TRAIL by Dox and were more pronounced in Mel-HO than in MeWo cells. SiRNA-mediated silencing of Mcl-1 resulted in a further significant decrease of cell viability and a further increase of apoptosis and necrosis in AdV-TRAIL-infected MeWo and Mel-HO cells. However, while in absolute terms, the effects were more pronounced in Mel-HO cells, in relative terms, they were stronger in MeWo cells. These results show that silencing of Mcl-1 represents a suitable approach to increase the cytotoxicity of a TRAIL-armed oncolytic adenovirus in melanoma cells.

**Key messages:**

• Cytotoxicity of TRAIL-expressing adenovirus can be enhanced by silencing of Mcl-1.

• The effect occurs in TRAIL-sensitive and TRAIL-resistant melanoma cells.

• Increase of apoptosis is the main mechanism induced by Mcl-1 silencing.

**Supplementary Information:**

The online version contains supplementary material available at 10.1007/s00109-021-02081-3.

## Introduction

The incidence of malignant melanoma, a highly aggressive skin cancer, has been increasing over the past few decades. Globally, each year, approximately 300,000 new patients are diagnosed with cutaneous melanoma, which thus belongs to the ten most common cancers in North America, Western Europe, and Oceania [1, 2]. Severe chemotherapy resistance and early dissemination of melanoma impeded effective therapy for decades. Only recently, selective inhibitors for the MAP kinases BRAF and MEK, as well as immune checkpoint modulators, as antibodies for CTLA4, PD1, or PDL1, have significantly improved melanoma therapy [3–5]. Although these new treatments significantly prolong overall survival of patients with metastatic melanoma, no adequate therapy is available yet able to cope with cancer recurrence and therapy resistance [5].

Apoptosis deficiency plays a major role in therapy resistance in cancers such as melanoma. Apoptosis can be induced via intrinsic and extrinsic apoptosis pathways. The latter are induced by death ligands as TRAIL and CD95L/Fas ligand [6, 7]. Death ligands bind to cognate death receptors [8] which results in activation of proapoptotic initiator caspase-8 and caspase-10 [8, 9]. In the next step, initiator caspases proteolytically cleave and activate effector caspases, such as caspase-3, caspase-6, and caspase-7, which themselves cleave a large number of death substrates to induce apoptosis [10]. Extrinsic apoptosis pathways induced by TRAIL or CD95L/Fas ligand may be further linked to the intrinsic apoptosis pathways via caspase-8-mediated processing of the proapoptotic BH3-only protein Bid, which may mediate activation of the multi-domain, proapoptotic Bcl-2 proteins Bax and Bak. These proteins increase mitochondrial permeability, thus inducing the release of proapoptotic mitochondrial factors, such as cytochrome c and second mitochondria-derived activator of caspases (Smac) [8]. Cytosolic cytochrome c triggers formation of the apoptosome, a multiprotein complex containing the adapter protein Apaf-1. Here, another initiator caspase, caspase-9, is activated, which strengthens the activation of effector caspase-3 [8]. As an antagonist of the cellular inhibitors of apoptosis proteins (cIAPs), Smac also drives effector caspase activation [11].

Due to its central role in apoptosis induction, as well as the fact that TRAIL induces apoptosis only in cancer cells, while normal cells are largely unaffected [12], the therapeutic employment of TRAIL has come into focus of cancer therapy. However, despite encouraging results observed in an experimental setting, the efficiency of this approach in patients remained only limited [13], most likely because cancer cells may be resistant to TRAIL per se or rapidly acquire inducible resistance upon TRAIL treatment [8, 14]. In melanoma cells, direct caspase activation by death ligands via the extrinsic apoptosis pathway appears less active. Thus, the mitochondrial activation loop via caspase-8 and Bid seems to be of particular importance [8]. However, the intrinsic apoptosis pathway is strictly controlled by prosurvival antiapoptotic Bcl-2 proteins and these proteins are often overexpressed in tumor cells and contribute to tumorigenesis and drug resistance [15, 16]. Mcl-1 belongs to the antiapoptotic Bcl-2 proteins. Over the last few years, this protein has received particular attention as a target for cancer therapy due to the fact that it is often overexpressed in cancer cells and that the MCL1 locus is one of the most frequently amplified regions of the human genome across a wide variety of cancers [17, 18]. Mcl-1 can heterodimerize and thus antagonize the proapoptotic activity of a number of proapoptotic Bcl-2 proteins, i.e., the BH3-only proteins Bim, tBid, Puma, and Noxa [19] as well as the proapoptotic, multi-domain proteins Bak and, to a lesser extent, Bax. Due to the neutralization of a broad spectrum of proapoptotic Bcl-2 proteins, Mcl-1 may compensate for the loss of other antiapoptotic Bcl-2 proteins in tumor cells, suggesting it as a potential key regulator of antiapoptotic control in cancer cells.

**Fig. 1:**
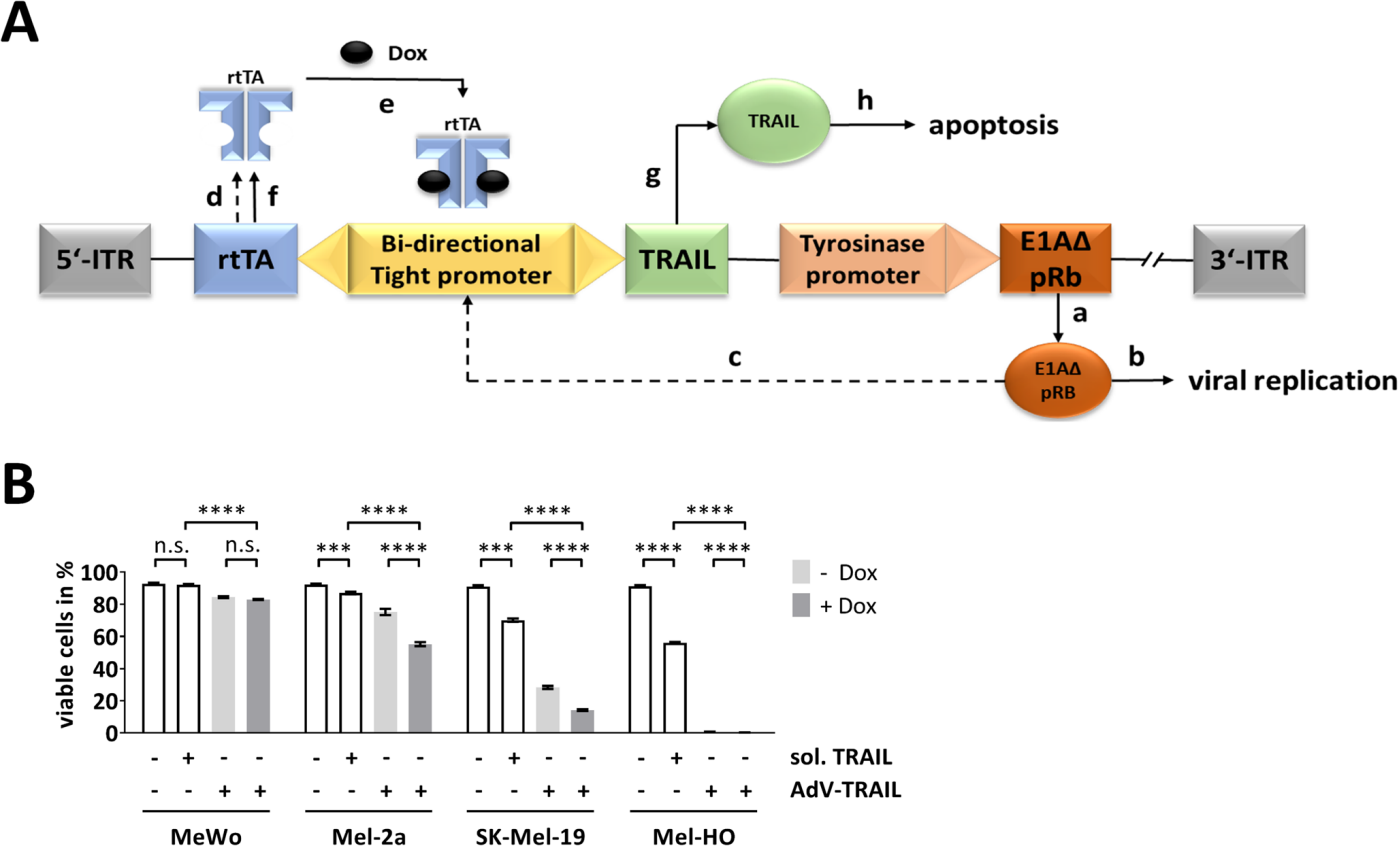
Resistance of certain melanoma cell lines to AdV-TRAIL treatment. **A** Schematic overview of the regulation elements of AdV-TRAIL. E1A lacking the retinoblastoma-binding domain (E1A_ΔpRb_) is controlled by a tyrosinase promoter which ensures a selective expression (a) and subsequent viral replication (b) in melanoma cells. E1A_ΔpRb_ also slightly transactivates the bi-directional doxycycline (Dox)-inducible Tight promoter (c) leading to minimal expression of the reverse tetracycline transactivator (rtTA) (d). In the presence of Dox, rtTA is able to bind to the Tight promoter (e) and induces strong rtTA expression (f), resulting in an activation loop. In parallel, activation of the Tight promoter in the presence of Dox leads to TRAIL expression (g), which subsequently induces apoptosis (h). In cells without tyrosinase promoter activity, E1A_ΔpRb_ and TRAIL are not expressed. Adenoviral inverted terminal repeats are labeled as 5′-ITR and 3′-ITR. **B** Cell viability of four melanoma cell lines after treatment with TRAIL and AdV-TRAIL. Cells were either treated with soluble TRAIL (50 ng/ml) or transduced with AdV-TRAIL (5 MOI). In the latter case, TRAIL expression was induced by Dox as indicated. Cell viability was detected by calcein-AM staining 48 h after transduction with AdV-TRAIL or treatment with soluble TRAIL, respectively. The mean percentages of calcein-AM-positive cells (viable) ± SEM of three independent experiments are shown. Statistical significance: *p < 0.05, **p < 0.01, ***p < 0.001, and ****p < 0.0001

Oncolytic adenoviruses (oAdV) selectively replicate in tumor cells, leading to their destruction [20]. Their safety was demonstrated in various animal cancer models in vivo as well as in clinical trials. However, clinical investigations revealed that first-generation oAdV were not sufficient to significantly alter the course of cancer progression in patients [21]. One way to increase the efficacy of oAdV was achieved by arming these viruses with cell death-promoting transgenes. Particularly, transgenes targeting apoptosis pathways were considered, and the proapoptotic death ligand TRAIL appeared to be the most promising candidate. When expressed from oAdV, TRAIL significantly increased the efficiency of virotherapy, as shown in several in vivo studies [22, 23]. Previously, we have developed AdV-TRAIL (Fig. 1A), an oAdV, which selectively expresses TRAIL and replicates in melanoma cells [24]. The virus showed an increased oncolytic activity in melanoma in vitro and in vivo resulting from TRAIL-mediated induction of apoptosis. Nevertheless, our investigations revealed that TRAIL resistance may limit the oncolytic efficacy also of AdV-TRAIL [24].

Here, we show that the cytotoxicity of AdV-TRAIL can be significantly enhanced in TRAIL-sensitive and TRAIL-resistant melanoma cells by silencing of Mcl-1. Enhancement of apoptosis by Mcl-1 silencing was determined as a key mechanism leading to increased cytotoxicity of AdV-TRAIL.

## Methods

### Cell culture

Origin of the four human melanoma cell lines used in this study (MeWo, Mel-2a, SK-Mel-19 and Mel-HO) was described previously [25]. All cell lines have significant tyrosinase mRNA expression [25, 26]. Human embryonal kidney (HEK-293) cells were used for adenovirus amplification and quantification. All cell lines were cultivated in Dulbecco’s modified Eagle’s medium (DMEM, 4.5 g/l glucose, Biowest, Nuaillé, France), 10% fetal calf serum (c.c.pro, Oberdorla, Germany) and 1% of each penicillin and streptomycin (Biowest, Nuaillé, France) at 37 °C and 5% CO_2_. Soluble TRAIL (Adipogen, San Diego, CA, USA) was added to the cell culture in a concentration of 50 ng/ml.

### Cell transfection with siRNA

Cells were reversely and transiently transfected with siRNA using Lipofectamine RNAiMAX (Thermo Fisher Scientific, Waltham, MA USA), according to the manufacturer’s protocol. For reverse transfection 15 pmol siRNA and 1 μl Lipofectamine were added to 500 μl cell suspension (10^5^ cells) prior to seeding in a 24-well plate. The siRNA (siMcl-1) which enables efficient silencing of Mcl-1 [27] 5′-AUAAUCUCCAGCGACUGCCdTdT-3′ and the control siRNA (siCon) [28] 5′-ACGUGACACGUUCGGAGAAdTdT-3′ were described previously. SiCon does not match any sequence present in the viral or human genome [28]. Both siRNAs were ordered from Eurofins Genomics (Ebersberg, Germany).

### Production of AdV-TRAIL and induction of TRAIL expression

AdV-TRAIL [24] is a E1/E3-deleted oncolytic adenovirus armed with TRAIL which is controlled by a Dox-inducible promoter (Figure 1A). Melanoma cell-selective replication of AdV-TRAIL is ensured by controlling an engineered adenoviral E1A protein lacking the retinoblastoma-binding domain (E1A_ΔpRb_) by a human tyrosinase promoter that has high selectivity for melanoma cells [29]. AdV-TRAIL was reamplified on HEK293 cells, purified by double CsCl density gradient centrifugation and the viral titer determined by standard plaque assay on HEK293 cells. The expression of TRAIL from AdV-TRAIL was induced by addition of Dox (Sigma-Aldrich, Darmstadt, Germany) to the cell culture medium at a concentration of 1 μg/ml. If the investigation period was longer than 24 h, Dox was re-applied at the same concentration after 24 h.

### Determination of AdV-TRAIL titers

AdV-TRAIL-infected melanoma cells were lysed by three freeze–thaw cycles and cell debris was removed by centrifugation at 6000×*g* for 10 min. To determine the number of infectious virus particles, confluent HEK293 cells, seeded in 24-wells, were incubated with 200 μl of the supernatant for 2 h. In parallel, HEK293 cells were infected at a MOI of 10, 1, 0.1, and 0.01 of AdV-TRAIL. These samples were used to generate a standard graph to calculate virus concentration of the investigated samples. Infected cells were washed with PBS to remove unbound virus and lysed in 100 μl PBS by three freeze–thaw cycles. Following centrifugation at 6000×*g* for 10 min, an aliquot of 5 μl was diluted 1:10 in PBS and inactivated at 95 °C for 5 min. Subsequently, 1.5 μl of the solution were used in a real-time PCR to determine the adenovirus copy number as previously described [30].

### Determination of cell viability

Cells (10^5^) were reversely transfected with 30 nM of siCon or siMcl-1 and seeded in 24-well plates. After 48 h, the cells were incubated with soluble TRAIL (50 ng/ml) or infected with AdV-TRAIL. Dox (1 μg/ml) was added, when TRAIL expression was intended. Cell viability was determined by calcein-acetoxymethyl (calcein-AM) staining at 24 h and 48 h after transduction with AdV-TRAIL*.* For measurement of cell viability, cells were harvested by trypsinization and stained with 2.5 μg/ml calcein-AM (PromoCell, Heidelberg, Germany) for 1 h at 37 °C. Subsequently, the labeled cells were washed with PBS and the percentage of calcein-AM-positive cells measured by flow cytometry (FL2H) with a FACS Calibur (BD Biosciences, Bedford, MA, USA).

### Cell killing assay

Cells (10^5^) were reversely transfected with 30 nM of siCon or siMcl-1 and seeded in 24-well plates. After 48 h, the cells were infected with AdV-TRAIL at a MOI of 2 (Mel-HO) and 25 (MeWo). Dox (1 μg/ml) was added when TRAIL expression was intended. Attached cells were stained at 24 h (Mel-HO) and 48 h (MeWo) after transduction with AdV-TRAIL. For staining, the medium was removed, and the cells were washed with PBS. To fix the cells, 200 μl 10 % trichloroacetic acid (Carl Roth, Karlsruhe, Germany) was added for 10 min. The cells were washed twice with PBS and overlaid with 200 μl crystal violet solution (0.1 % in 1 % EtOH, Carl Roth). After an incubation of 5 min, the cells were washed with PBS several times. The plate was allowed to dry overnight and photographed.

### Determination of apoptosis

Cell cycle analysis by propidium iodide labeling and quantification of apoptosis were performed as described previously [31]. Cells grown and treated in 24-well plates were harvested by trypsinization. The harvested cells were lysed in hypotonic buffer and the isolated nuclei were stained for 1 h with 40 μg/ml propidium iodide (Sigma-Aldrich). Cells in different cell cycle phases were quantified based on their DNA content by flow cytometry (FL3A) with a FACS Calibur (BD Biosciences).

### Determination of necrosis

Necrosis was determined by measuring lactate dehydrogenase (LDH) activity in cell culture supernatant using the CyQUANT™ LDH Cytotoxicity Assay according to the manufacturer’s protocol (Thermo Fisher Scientific). Cells were grown and treated in 24-well plates, as described above.

### Western blot analysis

Protein extraction and western blot analysis were performed as described previously [25]. Protein levels were quantified using ImageJ and normalized to β-actin levels. For detection of proteins in the supernatant, the conditioned media and cell debris were separated by centrifugation. The collected supernatant was subsequently concentrated using Amicon Ultra Centrifugal Filters (UFC200324, Merck, Darmstadt, Germany) and western blot analysis were performed. Primary antibodies used were E1A (sc-58658, Santa Cruz Biotechnology, Dallas, TX, USA; diluted 1:1000), TRAIL (3219, Cell Signaling Technology, Danvers, MA, USA; diluted 1:1000), Mcl-1 (4572, Cell Signaling; diluted 1:1000), caspase-8 (9746, Cell Signaling, 1:1000), cleaved caspase-3 (9664, Cell Signaling, 1:1000), cleaved caspase-9 (20750, Cell Signaling, 1:1000), and β-actin (3700, Cell Signaling; 1:1000). As secondary antibodies horseradish peroxidase-labeled goat anti-rabbit and goat anti-mouse immunoglobulins were used (Agilent Dako, Santa Clara, CA, USA; diluted 1:5000). Proteins were detected using Chemiluminescence HRP Reagent (Serva, Heidelberg, Germany) and visualized by the ChemiDoc™ MP Imaging System (Biorad, Feldkirchen, Germany).

### Statistical analyses

All data was collected in duplicates or triplicates, in at least three independent experiments. Mean values, SEMs, and statistical significance were calculated by enclosing all individual values of the independent experiments. Statistical significance was proven by Student’s *t* test (two-tailed, unpaired) using the GraphPad Prism 6 software. A *p* value of < 0.05 was considered statistically significant.

## Results

### Sensitivity of melanoma cells to TRAIL and AdV-TRAIL

Previous in vitro investigations have shown that melanoma cells have different sensitivity to TRAIL and TRAIL-armed oncolytic viruses ranging from high sensitivity to nearly complete resistance [24, 32, 33]. To investigate whether Mcl-1 silencing can overcome AdV-TRAIL resistance, we initially examined four melanoma cell lines (MeWo, Mel-2a, SK-Mel-19, Mel-HO) for their sensitivity to TRAIL/AdV-TRAIL. Soluble TRAIL was added to cells at a dose of 50 ng/ml and AdV-TRAIL at a MOI of 5. The expression of TRAIL in AdV-TRAIL is controlled by a Dox-inducible promoter (Fig. 1A). Therefore, AdV-TRAIL-infected cells received Dox to induce TRAIL expression or were left without Dox to investigate the cells response in the absence of TRAIL. Sensitivity of cells was determined by measuring cell viability using a calcein-AM flow cytometry assay 48 h after treatment with TRAIL or AdV-TRAIL. The cell line Mel-HO was sensitive to soluble TRAIL, whereas MeWo was completely resistant. Mel-2a showed low sensitivity to soluble TRAIL, while SK-Mel-19 cells were more sensitive than Mel-2a cells but less sensitive than Mel-HO cells (Fig. 1B). In all cell lines, AdV-TRAIL reduced cell viability significantly stronger than soluble TRAIL. Mel-HO cells were highly sensitive to AdV-TRAIL, whereas SK-Mel-19 and Mel-2a were moderately sensitive. The effects were already seen in the absence of Dox but were more pronounced after induction of TRAIL expression by addition of Dox, demonstrating that expression of TRAIL enhances the cytotoxicity of AdV-TRAIL. In clear contrast, the cytotoxicity of AdV-TRAIL in MeWo was only weak, and in particular, there was no difference between Dox-mediated TRAIL induction and no induction.

Because of the markedly different degree of sensitivity to TRAIL and AdV-TRAIL, the sensitive cell line Mel-HO, as well as the resistant cell line MeWo, were investigated further.

### Efficient downregulation of Mcl-1 by siRNA

**Fig. 2:**
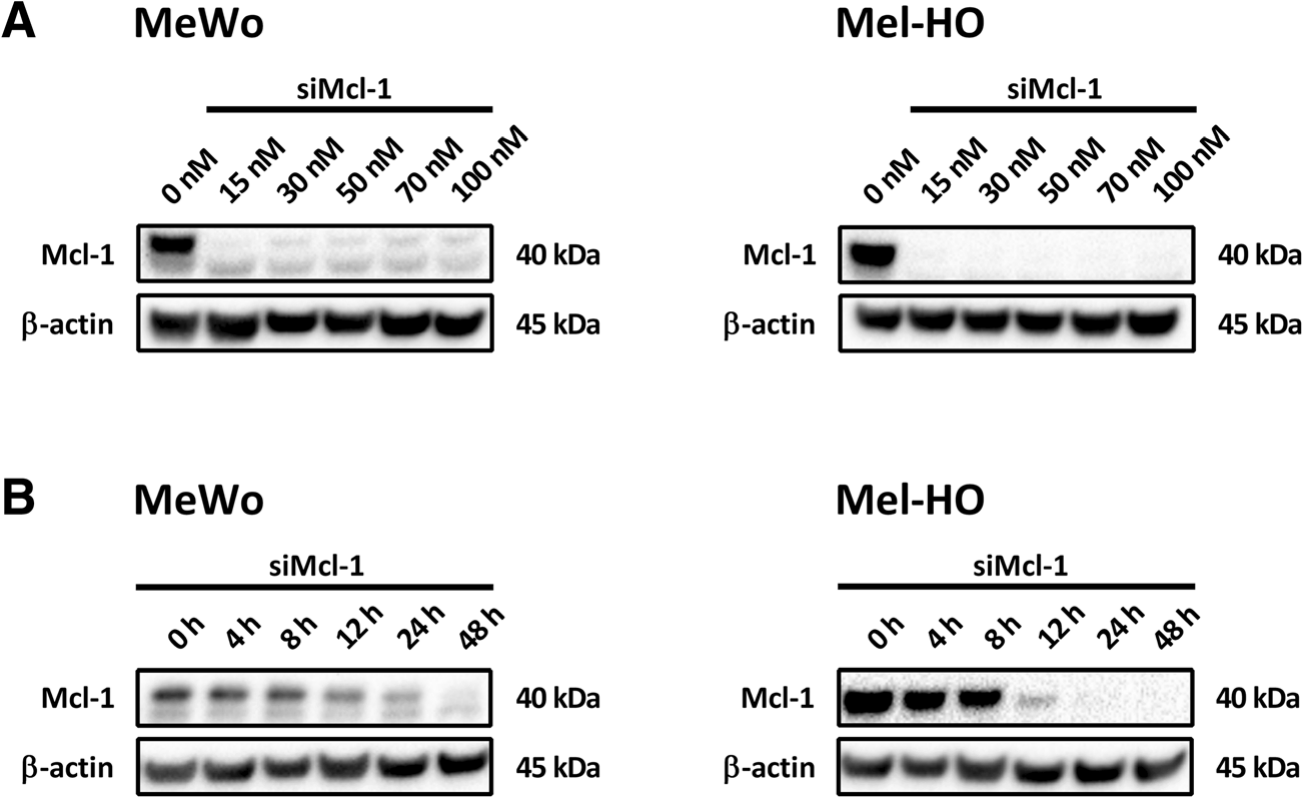
Efficient downregulation of Mcl-1 by Mcl-1 siRNAs in melanoma cells. Western blot analyses of Mcl-1 in melanoma cell lines MeWo and Mel-HO transfected with a siRNA targeting Mcl-1 (siMcl-1). **A** The levels of Mcl-1 were assessed 24 h after transfection with different concentrations of siMcl-1. **B** Cells were transfected with 30 nM siMcl-1 and Mcl-1 levels were analyzed after the indicated periods of time. Per lane, 30 μg protein were loaded and consistent blotting was proven by β-actin staining. Experiments were performed twice and gave very similar results

According to our previous data showing that silencing of Mcl-1 sensitizes melanoma cell lines to TRAIL [34], we hypothesized that Mcl-1 silencing may also increase the cytotoxicity of AdV-TRAIL in melanoma cells. To investigate this, we first sought the best conditions for silencing Mcl-1 in the cell lines MeWo and Mel-HO. The cell lines were transiently transfected with 15 to 100 nM of a siRNA directed against Mcl-1 (siMcl-1) [27], and Mcl-1 protein levels were determined by Western blot analysis 24 h later (Fig. 2A). Complete ablation of Mcl-1 in Mel-HO and nearly complete ablation in MeWo was seen already with the lowest siMcl-1 concentration of 15 nM. To investigate the Mcl-1 knockdown over time, both cell lines were transiently transfected with 30 nM siMcl-1, and Mcl-1 expression was determined by Western blotting up to 48 h later (Fig. 2B). A strong decrease in Mcl-1 expression was seen in Mel-HO cells already 12 h after siMcl-1 transfection, while almost complete downregulation was found after 24 h. In MeWo, weak silencing was seen 12 h after siMcl-1 transfection, whereas a nearly complete knockdown was seen 48 h after transfection. To ensure that Mcl-1 was strongly silenced in subsequent experiments, siMcl-1 was used at a concentration of 30 nM, and cells were incubated for 48 h before treatment with AdV-TRAIL.

### Mcl-1 silencing remains without effect on expression of adenoviral proteins and TRAIL as well as on replication of AdV-TRAIL

**Fig. 3:**
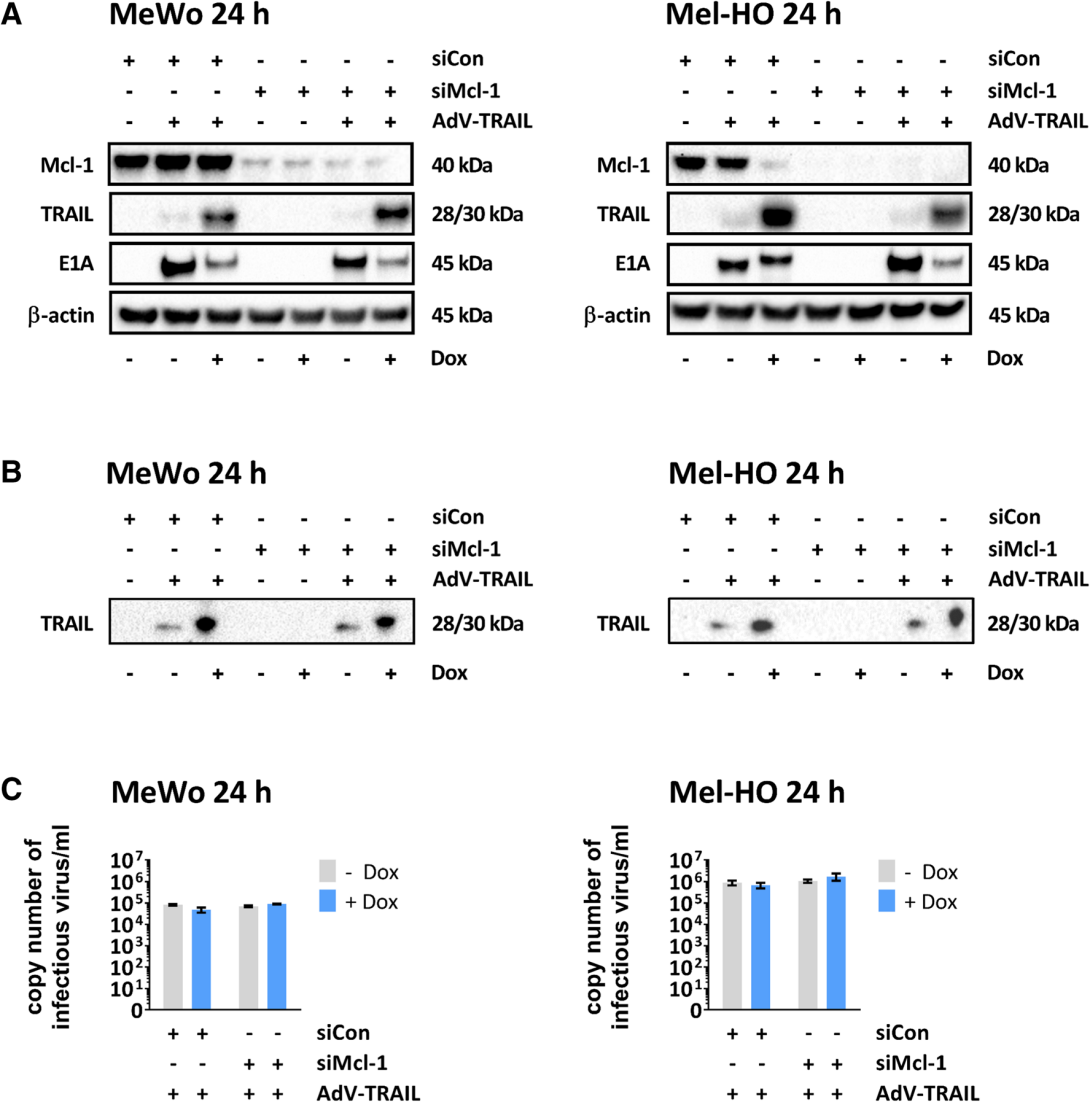
AdV-TRAIL replication and expression of E1A_ΔpRb_ and TRAIL in melanoma cells. **A** Western blot analyses of E1A_ΔpRb_, TRAIL, and Mcl-1 in the melanoma cell lines MeWo and Mel-HO. The cells were transfected with 30 nM of a control siRNA (siCon) or siMcl-1 and infected 48 h later with AdV-TRAIL at a MOI of 5 (MeWo) and 2 (Mel-HO). Simultaneously, TRAIL expression was induced by Dox as indicated. Proteins were extracted 24 h after infection and expression of Mcl-1, TRAIL, and E1A_ΔpRb_ was determined. As an internal loading control, β-actin was used. Representative data from three independent experiments are shown. **B** For detection of TRAIL in the supernatant the same Western blot assay was repeated. Supernatant was collected 24 h after infection, concentrated and the expression of TRAIL was determined. Representative data from two independent experiments are shown. **C** Replication of AdV-TRAIL. To measure viral replication of AdV-TRAIL MeWo and Mel-HO cells were treated as described above, except both cell lines were infected with AdV-TRAIL at a MOI of 2. To quantify genome copy numbers of infectious virus particles, the cells were lysed 24 h after viral infection by freeze–thaw cycles, centrifuged and the supernatant used for infection of HEK293 cells for 2 h. After washing, the cells were lysed and the amount of viral DNA was quantified by real-time PCR. Presented are the mean genome copy numbers ± SEM of three independent experiments

We next examined whether Mcl-1 silencing affects the expression of TRAIL and adenoviral E1A, which is the central viral regulator protein of adenoviral replication. In the previous experiment, application of AdV-TRAIL at a MOI of 5 resulted in strong induction of cell lysis within 48 h (Fig. 1B) making a realistic measurement of TRAIL and E1A expression uncertain under the same experimental conditions. Therefore, here, we reduced the investigation time and the dose of AdV-TRAIL when Mel-HO cells were infected. Both MeWo and Mel-HO cells were first transfected with siMcl-1 for 48 h and subsequently infected with AdV-TRAIL at a MOI of 5 (MeWo) or a MOI of 2 (Mel-HO) and expression of Mcl-1, E1A, and TRAIL were examined by western blotting 24 h later in the presence and in the absence of Dox (Fig. 3A, B, Supplementary Figure S1), while, as expected, the expression of TRAIL both in the cells and their supernatant was strongly induced by Dox, surprisingly E1A expression was slightly reduced in the presence of Dox. The reason is not clear. Dox is known to be able to suppress the expression of cellular transcription factors [35] and a similar mechanism may be involved in suppression of adenoviral E1A. TRAIL was also detected at low levels in the absence of Dox, which indicates some leakiness of the Dox-inducible promoter. Cellular Mcl-1 expression remained unaffected in AdV-TRAIL-infected MeWo cells, whereas we observed strong Mcl-1 downregulation in AdV-TRAIL-infected Mel-HO cells when TRAIL was induced by Dox. Transfection with siMcl-1 resulted in strong inhibition of cellular Mcl-1 expression in AdV-TRAIL-infected MeWo cells and nearly complete ablation of Mcl-1 expression in AdV-TRAIL-infected Mel-HO cells. Furthermore, silencing of Mcl-1 had no significant effect on the expression of adenoviral E1A and TRAIL expression, as compared to cells transfected with siCon (Fig. 3A).

We next examined virus replication in siMcl-1-transfected and AdV-TRAIL-infected MeWo and Mel-HO cells. AdV-TRAIL was administered at a MOI of 2 in each cell line, and the levels of replicating virus were determined by real-time PCR. The virus levels of AdV-TRAIL were higher in Mel-HO cells than in MeWo cells (1 × 10^6^ virus genome copies/ml vs. 7 × 10^4^ virus genome copies/ml), but remained unaffected by Mcl-1 silencing and/or TRAIL expression (Fig. 3B).

These results demonstrate that Mcl-1 silencing did not affect AdV-TRAIL replication nor virus-mediated TRAIL expression in either cell line. The results also show that AdV-TRAIL downregulates Mcl-1, but only in Mel-HO cells after induction of TRAIL by Dox.

### Mcl-1 silencing increases cytolytic activity of AdV-TRAIL in TRAIL-resistant melanoma cells

**Fig. 4:**
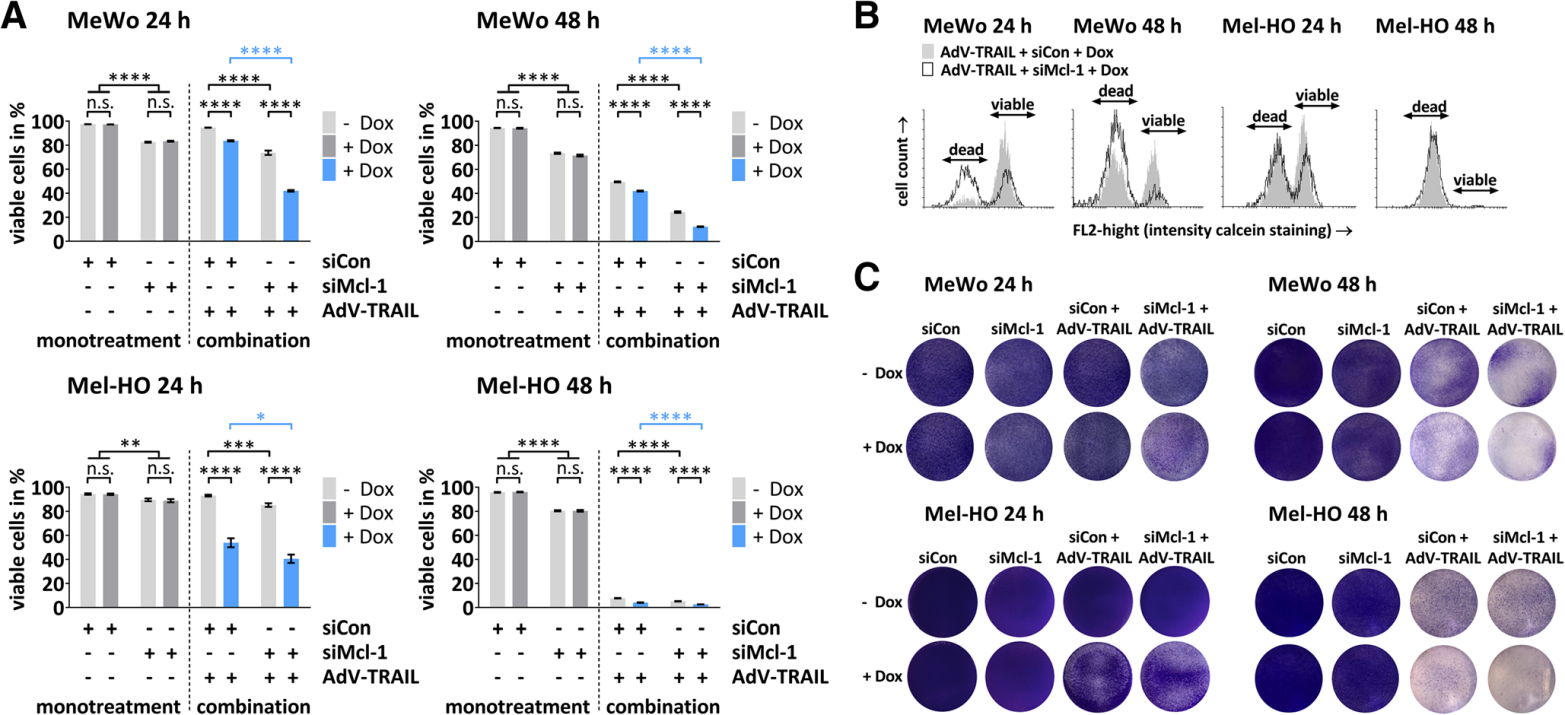
Viability of melanoma cells after treatment with AdV-TRAIL and siMcl-1. **A** Viability of MeWo and Mel-HO cells after treatment with AdV-TRAIL and siMcl-1. MeWo and Me-HO cells were transfected with 30 nM of siMcl-1 or siCon and infected 48 h later with AdV-TRAIL at a MOI of 25 (MeWo) or 2 (Mel-HO). TRAIL expression was induced by Dox as indicated. Cell viability was determined by calcein-AM staining and flow cytometry 24 h and 48 h after infection with AdV-TRAIL. Data are expressed as mean percentages of calcein-AM-positive cells (viable) ± SEM of three independent experiments. Statistical significance: *p < 0.05, **p < 0.01, ***p < 0.001, and ****p < 0.0001. **B** The histograms show the amount of viable and dead cells in the indicated samples. **C** Cell killing assays of Mel-HO and MeWo melanoma cell lines. Cells were treated as described in **A** and crystal violet staining of attached cells was performed at 48 h (MeWo) and 24 h (Mel-HO) after transduction with AdV-TRAIL

To investigate, whether Mcl-1 silencing increases the cytolytic activity of AdV-TRAIL, we transfected MeWo and Mel-HO cells with siMcl-1 or siCon. After 48 h, cells were infected with AdV-TRAIL at a MOI of 2 (Mel–HO) or 25 (MeWo). We increased the AdV-TRAIL dose for infection of MeWo in this experiment as an initial experiment showed that effects of Mcl-1 silencing were significant but weak using a MOI of 5 (Supplementary Figure S2A, B). Cell viability was determined by flow cytometry after calcein-AM staining at 24 h and 48 h after infection. Already Mcl-1 silencing alone resulted in a reduction of cell viability down to 80 % in Mel-HO and down to 72 % in MeWo at 48 h (Fig. 4A). Infection of MeWo cells with AdV-TRAIL reduced the viability to 50% in the absence of Dox and to 42%, when TRAIL was induced by Dox after 48 h (Fig. 4A). The further increase of cell toxicity by addition of Dox seems to result from high expression levels of TRAIL induced by use of an MOI of 25 of AdV-TRAIL, as Dox application had no effect on cell viability when AdV-TRAIL was used at an MOI of 5 (Fig. 1B, Supplementary Figure S2A). In Mel-HO, the effects of AdV-TRAIL on cell viability were even more pronounced. Only 8% of Mel-HO cells remained alive at 48 h after infection with AdV-TRAIL. This percentage was further decreased to 4% when TRAIL was induced by Dox (Fig. 4A). Silencing of Mcl-1 in AdV-TRAIL-infected cells resulted in significantly enhanced cytotoxicity of AdV-TRAIL. In MeWo, cell viability dropped to 25 % and in Mel-HO to 5 % in the absence of Dox 48 h after infection (Fig. 4A, B). After induction of TRAIL by Dox, cell viability significantly decreased further to 12% in MeWo and to 3% in Mel-HO (Fig. 4A, B). Cell killing assays confirmed the loss of cell viability by the treatments (Fig. 4C). Based on the data from Fig. 4A, we calculated the relative increase of cytotoxicity mediated by Mcl-1 silencing in AdV-TRAIL-infected melanoma cells with Dox-induced TRAIL expression 48 h after application of the virus. Co-silencing of Mcl-1 increased the loss of viability by 71% in MeWo but only by 36% in Mel-HO cells compared to treatment with AdV-TRAIL alone.

These results indicate that in both cell lines cytotoxicity of AdV-TRAIL can be strongly enhanced by co-silencing of Mcl-1. However, in relative terms, this effect was distinctly stronger in MeWo than in Mel-HO cells.

### Mcl-1 silencing increases apoptosis induction by AdV-TRAIL in TRAIL-resistant melanoma cells

**Fig. 5:**
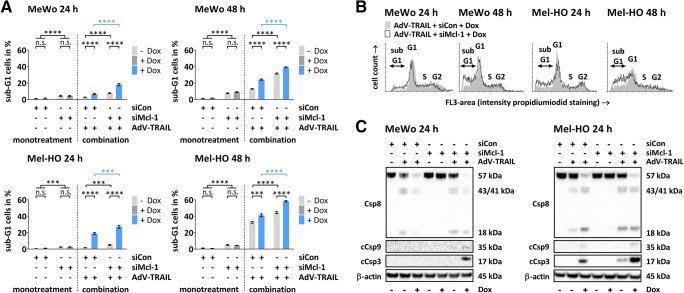
Apoptosis induction and caspase-3 activation in melanoma cells treated with AdV-TRAIL and siMcl-1. **A** Apoptosis induction in MeWo and Mel-HO cells after treatment with AdV-TRAIL and siMcl-1. Cells were treated as described in Fig. 4. Apoptosis was determined by propidium iodide staining and flow cytometry at 24 h and 48 h after transduction with AdV-TRAIL. The mean percentages of sub-G1 (apoptotic) cells ± SEMs of three independent experiments are shown. Statistical significance: *p < 0.05, **p < 0.01, ***p < 0.001, and ****p < 0.0001. **B** The histograms show the amount of apoptotic sub-diploid (sub-G1) cells as well as cells in cell cycle phases gap 1 (G1), gap 2 (G2), and synthesis (S) in the indicated samples. **C** Western blot analyses of caspase-8 (Csp8), cleaved caspase-9 (cCsp9), and cleaved caspase-3 (cCsp3). Cells were treated as described above and protein levels were examined 24 h after infection of the cells with AdV-TRAIL. Consistent blotting was verified by β-actin staining. Representative data from at least two independent experiments are shown

To determine whether the increase of cytotoxicity of AdV-TRAIL by Mcl-1 silencing was caused by an increase in apoptosis induction, the number of apoptotic cells in MeWo and Mel-HO cells was determined by cell cycle analysis after propidium iodide staining and flow cytometry. Cells were treated in the same way as for the determination of cell viability. In untreated control cells, the number of apoptotic cells was 1%. Merely silencing Mcl-1 significantly increased apoptosis in both cell lines, with a slightly stronger effect in the MeWo. Nonetheless, the number of apoptotic cells did not exceed 10% (Fig. 5A). Also, treatment of MeWo cells with AdV-TRAIL increased the number of apoptotic cells, but the percentage of apoptotic cells remained below 15% (Fig. 5A, B), and even after induction of TRAIL by Dox, the percentage increased only up to 24% (Fig. 5A, B). In Mel-HO cells, the percentage of apoptotic cells was distinctly higher after infection with AdV-TRAIL. In the uninduced state up to 33% and after induction of TRAIL by Dox up to 41% of the cells were apoptotic (Fig. 5A, B). Silencing of Mcl-1 significantly increased apoptosis induction in AdV-TRAIL-infected MeWo and Mel-HO cells. In MeWo cells, the percentage of apoptotic cells increased to up to 32 and 39 % and in Mel-HO cells to up to 44 and 58% in the uninduced and Dox-induced state, respectively (Fig. 5A, B). Calculation of the average relative increase of apoptotic cells mediated by Mcl-1 silencing in AdV-TRAIL-infected melanoma cells with Dox-induced TRAIL expression 48 h after application of the virus revealed that co-silencing of Mcl-1 increased the percentage of apoptotic cells by 61% in MeWo but only by 31% in Mel-HO cells compared to the sole AdV-TRAIL treatment.

These results indicate that Mcl-1 silencing increases apoptosis in AdV-TRAIL-infected MeWo and Mel-HO cells. However, in relative terms, the induction of apoptosis was distinctly higher in MeWo than in Mel-HO cells.

### Silencing of Mcl-1 enhances caspase-3 activation in AdV-TRAIL-transduced TRAIL-resistant melanoma cells

In order to elucidate the mechanisms responsible for increased apoptosis induction in MeWo and Mel-HO cells after Mcl-1 silencing and AdV-TRAIL infection, the activation of the initiator caspase-8, the intrinsic initiator caspase-9, and the main effector caspase-3 were examined by western blot analysis 24 h after application of AdV-TRAIL. Following silencing of Mcl-1, no activation of caspase-3, caspase-8, or caspase-9 was detected. In contrast, as seen by detection of the 43, 41, and 18 kDa cleavage products of caspase-8, 35 kDa cleavage product of caspase-9, and 17 kDa cleavage product of caspase-3, there was strong activation of the respective caspases in Mel-HO cells after infection with AdV-TRAIL and induction of TRAIL expression by Dox (Fig. 5C, Supplementary Figure S1). Caspase-8 was also activated in Mel-HO cells when TRAIL expression was not induced (Fig. 5C, Supplementary Figure S1), suggesting that the leaky expression of TRAIL in the uninduced state (Fig. 3A) was sufficient to activate caspase-8. Silencing of Mcl-1 in AdV-TRAIL-treated Mel-HO cells led to a distinctly stronger activation of caspase-3, but not of caspase-8 and caspase-9. This effect was seen not only when TRAIL expression was induced by Dox but also when TRAIL expression was not induced. Here again, leaky TRAIL expression may contribute to the latter result. Similar to Mel-HO cells caspase-8 was activated in MeWo cells after infection with AdV-TRAIL due to leaky expression of TRAIL in the uninduced state (Fig. 3A) and more prominently after full induction of TRAIL expression by Dox (Fig. 5C, Supplementary Figure S1). However, in MeWo cells, activation of caspase-3 and weak activation of caspase-9 were only observed in AdV-TRAIL-infected cells with induced TRAIL expression when Mcl-1 was silenced.

These results indicate that Mcl-1 silencing enhances the activation of caspase-3 in AdV-TRAIL-infected cells, in particular when the expression of TRAIL was induced by Dox.

### Silencing of Mcl-1 enhances AdV-TRAIL-mediated necrosis in TRAIL-resistant melanoma cells

**Fig. 6:**
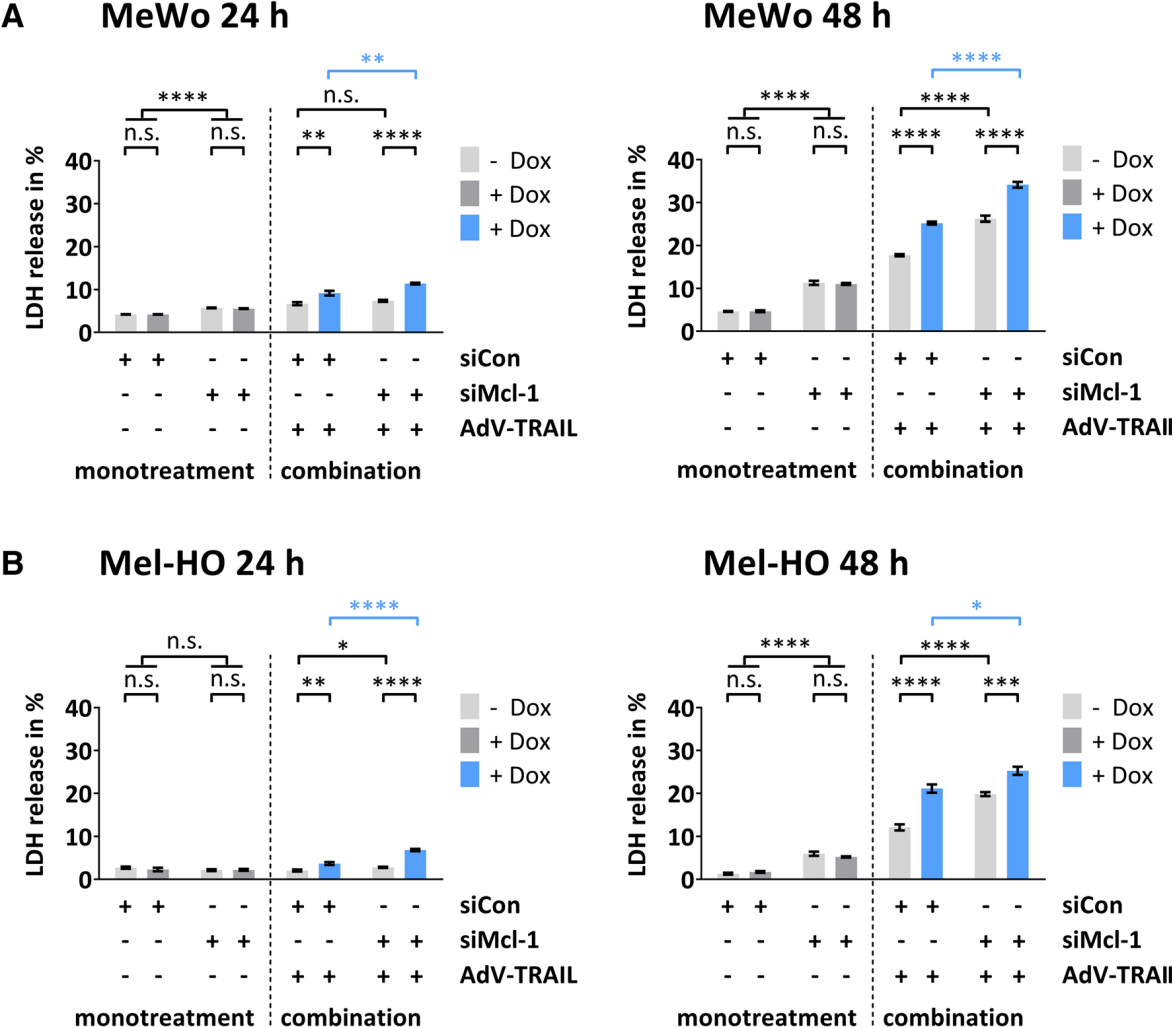
Necrosis induction in melanoma cells treated with AdV-TRAIL and siMcl-1. MeWo and Mel-HO cells were treated as described in Fig. 4. Lactate dehydrogenase (LDH) release was detected at 24 h and 48 h after infection with AdV-TRAIL. Cytotoxicity values are given as percentage of LDH values after complete cell lysis. Presented are the mean percentages of LDH release ± SEM of three independent experiments. Statistical significance: *p < 0.05, **p < 0.01, ***p < 0.001, and ****p < 0.0001

To determine whether the induction of necrosis may also contribute to the increase of cytotoxicity of AdV-TRAIL by Mcl-1 silencing, we next measured LDH release of MeWo and Mel-HO cells. In untreated cells, LDH release was below 5%, whereas silencing of Mcl-1 significantly increased LDH release, of both MeWo and Mel-HO cells (Fig. 6). Nevertheless, LDH release caused by Mcl-1 silencing did not exceed 11% in either cell line. Treatment of MeWo cells with AdV-TRAIL increased LDH release up to 18% and induction of TRAIL expression by Dox further increased LDH release up to 25%. In Mel-HO cells, administration of AdV-TRAIL resulted in a LDH release of 12 and 21% in the uninduced state and after induction of TRAIL by Dox, respectively. Silencing of Mcl-1 in AdV-TRAIL-infected MeWo and Mel-HO cells significantly increased LDH release in both cell lines to up to 26 and 34% in MeWo cells and to up to 20 and 25% in Mel-HO cells in the uninduced and Dox-induced state, respectively (Fig. 6).

These results indicate that necrosis induction induced by AdV-TRAIL is enhanced by silencing of Mcl-1.

## Discussion

Here, we show that cytotoxicity of the TRAIL-expressing oncolytic adenovirus AdV-TRAIL can be significantly improved in TRAIL-resistant and TRAIL-sensitive melanoma cell lines by silencing of the antiapoptotic Bcl-2 protein Mcl-1. Moreover, our investigations revealed enhanced induction of apoptosis via activation of caspase-3 as the primary mechanism contributing to the increased cytotoxicity of AdV-TRAIL by Mcl-1 silencing.

Although new treatments have improved overall survival rates of patients with melanoma, current therapies have not changed the fact that melanoma remains the major cause of skin cancer-related deaths [36]. Hence, new strategies to effectively combat melanoma are required. A promising new concept is represented by oncolytic viruses that selectively replicate in and destroy tumor cells. To improve the efficiency of oncolytic viruses, they can be equipped with death-promoting transgenes. In this regard, we showed that arming an oncolytic adenovirus with Dox-inducible expression of the death ligand TRAIL (AdV-TRAIL) selectively induced apoptosis in melanoma cells and significantly enhanced its antitumor efficiency [24]. However, therapeutic use of AdV-TRAIL is limited, as melanoma cells can be resistant to TRAIL [24, 37]. Consistent with this, we found here that AdV-TRAIL was highly cytotoxic in the TRAIL-sensitive melanoma cell line Mel-HO but was not very effective in the TRAIL-resistant melanoma cell line MeWo. Low susceptibility of the extrinsic apoptosis pathway for activation by TRAIL seems to play a central role for inability of TRAIL to kill melanoma cells. Therefore, activation of intrinsic apoptosis pathway is of particular importance. This pathway, however, is controlled by antiapoptotic Bcl-2 proteins, among them Mcl-1, which is a key regulator of antiapoptotic, prosurvival signaling in cancer cells. Mcl-1 was highly expressed in MeWo and Mel-HO cells and silencing it significantly increased AdV-TRAIL-induced cytotoxicity and made both AdV-TRAIL-infected melanoma cell lines more sensitive to TRAIL-induced apoptosis. Interestingly, these effects were also observed in Mel-HO, although in this cell line Mcl-1 was already strongly downregulated after infection with AdV-TRAIL and induction of TRAIL. The latter may be explained by the sensitivity of this cell line to TRAIL, as it has been shown that Mcl-1 can be cleaved at two aspartic acid residues (Asp127 and Asp157) by caspase-3, which itself is activated by TRAIL [38]. In accordance with this, caspase-3 was selectively upregulated in Mel-HO after AdV-TRAIL infection and induction of TRAIL by Dox. Our finding may be of importance for treatment of melanomas, as they demonstrate that silencing of Mcl-1 cannot only increase the cytotoxicity of the AdV-TRAIL in TRAIL-resistant melanoma cells but also in TRAIL-sensitive melanoma cells, even when Mcl-1 is expressed at a very low level.

Regarding the strength of the increase in cytotoxicity induced in AdV-TRAIL-infected melanoma cells after silencing of Mcl-1, this was more pronounced in MeWo than in Mel-HO. This indicates that inhibition of Mcl-1 has a greater impact on AdV-TRAIL activity when the melanoma cells are resistant to TRAIL. In accordance with this observation, we found that Mcl-1 knockdown had a stronger effect on apoptosis induction in AdV-TRAIL-infected MeWo compared to Mel-HO. Less susceptibility of Mel-HO may be explained by the fact that in this cell line, induction of apoptosis by the TRAIL-mediated extrinsic apoptosis pathway is already effective and a further increase in apoptosis by enhanced activation of the intrinsic apoptosis pathway may therefore be limited. In contrast, in TRAIL-resistant MeWo, apoptosis is only weakly induced via the TRAIL-mediated extrinsic pathway. Therefore, the removal of inhibitors of the intrinsic apoptosis pathway, as demonstrated here by silencing of Mcl-1, has greater effects on apoptosis induction.

Oncolytic adenoviruses usually kill tumor cells by necrosis-like cell death, which is independent of the basic apoptosis machinery of the infected cell [39]. Confirming this fact, we found in both cell lines AdV-TRAIL induced necrosis, even in the absence TRAIL induction. Interestingly, induction of TRAIL by Dox in AdV-TRAIL-infected MeWo and Mel-HO cells resulted in increased necrosis. Moreover, when AdV-TRAIL was combined with Mcl-1 silencing, a further significant increase of necrosis was detected. Previously, it has been shown in tumor cells that TRAIL can also induce necrosis via necroptosis [40]. Necroptosis describes a programmed form of necrosis, which is induced by activation of a signal cascade that involves the serine–threonine receptor interacting protein (RIP) kinases RIPK1 and RIPK3 [41, 42]. Induction of necroptosis is independent of caspase-activated apoptosis but can effectively be prevented by caspase-8. Even non-stimulated caspase-8 is sufficient for the suppression of programmed necrosis [43]. We found strong activation of caspase-8 in AdV-TRAIL-infected cells. Therefore, most likely secondary necrosis and not necroptosis is the basic mechanism leading to increased necrosis caused by TRAIL expression as well as by Mcl-1 silencing in AdV-TRAIL-infected melanoma cells.

We also investigated whether there is an induction of reactive oxygen species (ROS) following AdV-TRAIL and Mcl-1 treatment in both MeWo and Mel-HO cells. However, there were not any significant changes of ROS levels in response to AdV-TRAIL or siMcl-1 treatment (results not shown). Thus, induction of ROS does not appear as relevant for the effects of AdV-TRAIL or siMcl-1 described here in melanoma cells.

Increasing the cytotoxicity of AdV-TRAIL by silencing of Mcl-1 may have relevance for further employment of the virus in melanoma therapy. In particular, the effectiveness and the safety of our approach must now be confirmed in vivo. In the last few years, several small molecules have been developed which efficiently inhibit Mcl-1 in vivo [44, 45]*.* However, a clinical phase I dose-escalation study for treatment of multiple myeloma, non-Hodgkin lymphoma, or acute myeloid leukemia by the Mcl-1 inhibitor AMG 397 was terminated due to cardiac toxicity. It has been shown that Mcl-1 plays a crucial role in the survival of hematopoietic stem cells, lymphocytes and cardiomyocytes [46–48]. Thus, inhibition of Mcl-1 in normal cells by small molecule inhibitors may be the cause for the observed side effects. Such side effects could be avoided by tumor cell-specific inhibition of Mcl-1, for example by RNA interference-mediated silencing of Mcl-1. In this regard, we found that transiently expressed miR-193b [34] as well as anti-Mcl-1 artificial microRNAs and small hairpin RNAs are able to suppress Mcl-1 in melanoma cells (results not shown). These small regulatory RNAs can be easily inserted into the genome of AdV-TRAIL, in order to enable their melanoma cell-specific delivery and silencing of Mcl-1.

In conclusion, we show here that cytotoxicity of AdV-TRAIL can be enhanced in TRAIL-resistant and TRAIL-sensitive melanoma cells by co-silencing of the antiapoptotic Bcl-2 protein Mcl-1 by activation of apoptosis and necrosis. Targeting of Mcl-1 may therefore contribute to a more effective treatment of melanoma using TRAIL-expressing oncolytic adenovirus.

## Supplementary information

ESM 1 (DOCX 302 kb)

## Data Availability

All data generated or analyzed during this study are included in this published article and its supplementary information files.
