# Supplementary material for: Silencing of Mcl-1 overcomes resistance of melanoma cells against TRAIL-armed oncolytic adenovirus by enhancement of apoptosis
Source: J Mol Med (Berl). 2021 May 24;99(9):1279–91. doi: 10.1007/s00109-021-02081-3 (PMC8367928; doi:10.1007/s00109-021-02081-3)
Supplement: Supplementary file 1 — (DOCX 302 kb) [file 109_2021_2081_MOESM1_ESM.docx]

**Suppelements**


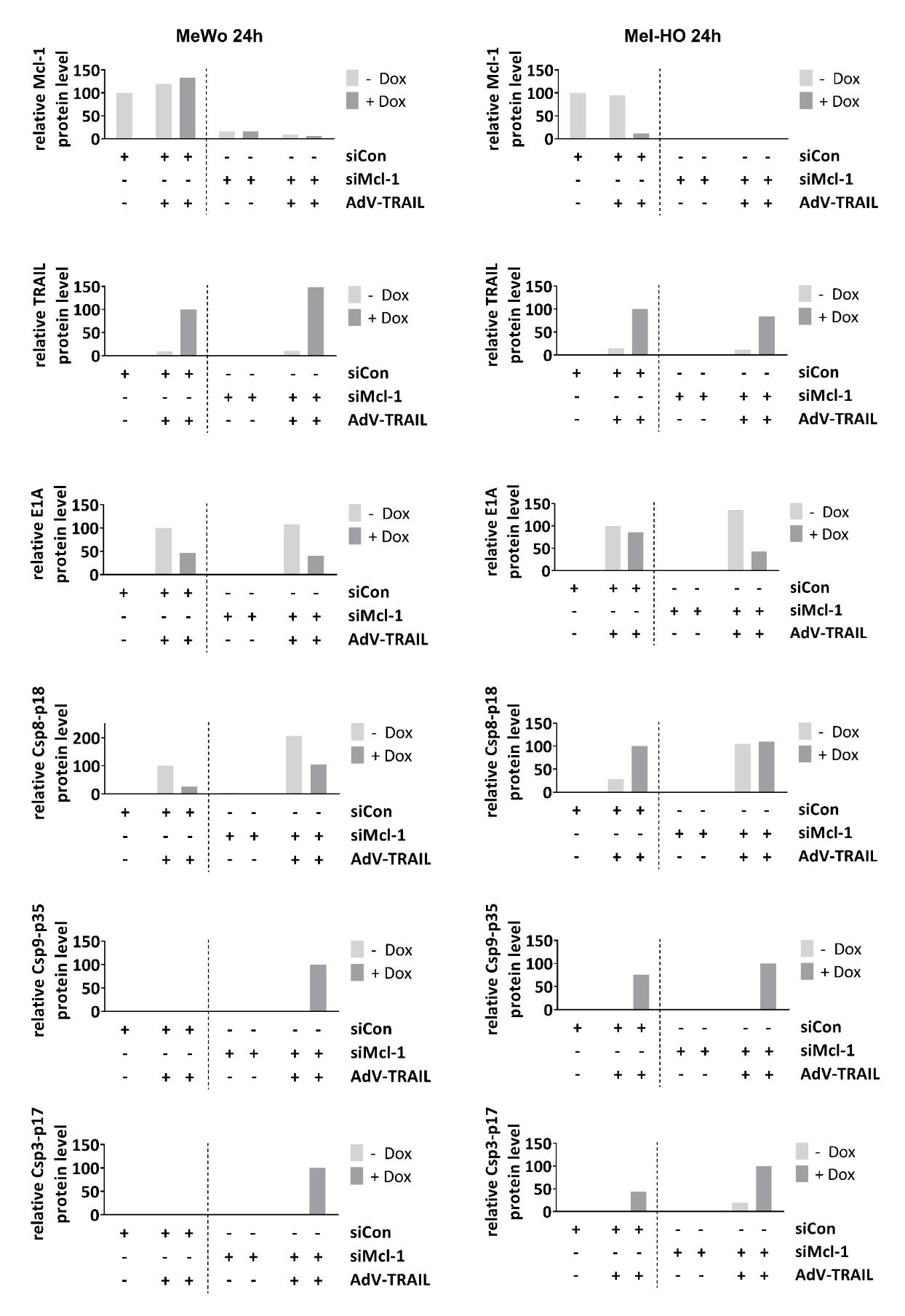
 **Figure S1. Quantifications of Westernblot signals from Fig. 3A and 5C.** Signal intentities are shown relative to β-actin.


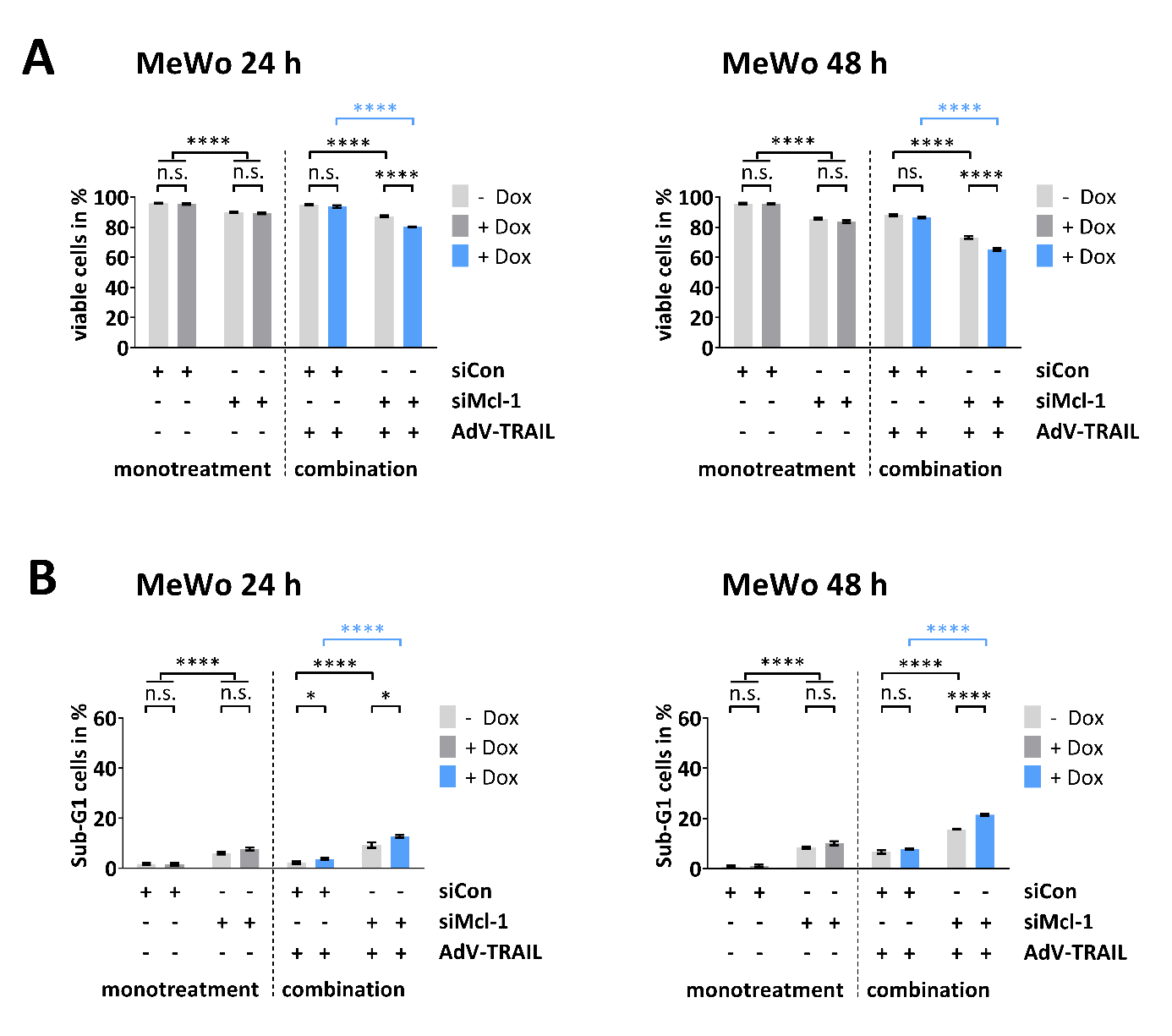


**Figure S2. Viability of and apoptosis induction in MeWo cells treated with AdV‑TRAIL and siMcl‑1.**

Cells were treated as described in Figure 4, but AdV-TRAIL was used at an MOI of 5. **(A)** Cell viability was determined by calcein‑AM staining and flow cytometry 24 h and 48 h after infection with AdV‑TRAIL. Data are expressed as mean percentages of calcein-AM positive cells (viable) ± SEM of three independent experiments. **(B)** Apoptosis was determined by propidium iodide staining and flow cytometry at 24 h and 48 h after transduction with AdV‑TRAIL. The mean percentages of sub-G1 (apoptotic) cells ± SEMs of three independent experiments are shown. Statistical significance: * p < 0.05, ** p < 0.01, *** p < 0.001 and **** p < 0.0001
